# Myosin-cross-reactive antigen (MCRA) protein from *Bifidobacterium breve *is a FAD-dependent fatty acid hydratase which has a function in stress protection

**DOI:** 10.1186/1471-2091-12-9

**Published:** 2011-02-17

**Authors:** Eva Rosberg-Cody, Alena Liavonchanka, Cornelia Göbel, R Paul Ross, Orla O'Sullivan, Gerald F Fitzgerald, Ivo Feussner, Catherine Stanton

**Affiliations:** 1 Alimentary Pharmabiotic Centre, Cork, Ireland; 2 Microbiology Department, University College Cork, Cork, Ireland; 3 Department Plant Biochemistry, Albrecht-von-Haller-Institute, Georg-August-University Göttingen, Göttingen, Germany; 4 Teagasc Food Research Centre, Moorepark, Fermoy, Co. Cork, Ireland

## Abstract

**Background:**

The aim of this study was to determine the catalytic activity and physiological role of myosin-cross-reactive antigen (MCRA) from *Bifidobacterium breve *NCIMB 702258. MCRA from *B. breve *NCIMB 702258 was cloned, sequenced and expressed in heterologous hosts (*Lactococcus *and *Corynebacterium*) and the recombinant proteins assessed for enzymatic activity against fatty acid substrates.

**Results:**

MCRA catalysed the conversion of palmitoleic, oleic and linoleic acids to the corresponding 10-hydroxy fatty acids, but shorter chain fatty acids were not used as substrates, while the presence of trans-double bonds and double bonds beyond the position C12 abolished hydratase activity. The hydroxy fatty acids produced were not metabolised further. We also found that heterologous *Lactococcus *and *Corynebacterium *expressing MCRA accumulated increasing amounts of 10-HOA and 10-HOE in the culture medium. Furthermore, the heterologous cultures exhibited less sensitivity to heat and solvent stresses compared to corresponding controls.

**Conclusions:**

MCRA protein in *B. breve *can be classified as a FAD-containing double bond hydratase, within the carbon-oxygen lyase family, which may be catalysing the first step in conjugated linoleic acid (CLA) production, and this protein has an additional function in bacterial stress protection.

## Background

The Streptococcal 67 kDa MCRA-like proteins represent a family of proteins which are present in a wide range of bacteria. It has been reported that some members of the MCRA-like proteins have fatty acid hydratase activity [1,2]. Recently, the MCRA protein from *Streptococcus pyogenes *M49 was reported to be a FAD-containing enzyme, which acts as hydratase on *cis*-9 (9Z) - and *trans*-11 (11E)-double bonds of C-16, C-18 non-esterified fatty acids producing 10-hydroxy and 10,13-dihydroxy fatty acids [1].

The expression of the *mcra *gene was found to be up-regulated by the stress responsive alternative transcription factor σ^B ^in *Staphylococcus aureus *[3]. It could be speculated that *in vivo *conditions such as high temperature at the site of infection, leading to stress in *S. aureus *may induce the stress responsive σ^B ^transcription factor [3]. It is a general belief that the MCRA protein is involved in virulence in *Staphylococcus *and *Streptococcus *[4]. A 67 kDa streptococcal MCRA protein was up-regulated in group A *Streptococcus *during experimental mouse soft tissue infection [5] and in the asymptomatic phase of infection in cynomolgus macaques monkeys infected with group A *Streptococcus *[6]. However, a *mcra *deletion mutant in *Streptococcus pyogenes *showed only mild alteration in virulence properties, such as blood survival, adherence and internalization to human keratinocytes [1]. Dunman *et al. *(2001) found the *mcra *gene was down-regulated by Agr, one of the most studied virulence response regulators in *Staphylococcus aureus*, along with other cell surface virulence factors [7]. Growth-phase associated regulation of the *mcra *gene was abrogated in a *rgg *mutant strain of *Streptococcus pyogenes *causing the mutant strain to synthesize the protein during both the exponential and stationary phases of growth, in contrast to the wild-type strain which only synthesized the protein in the exponential phase of growth. The transcriptional regulatory protein Rgg coordinates amino acid catabolism and virulence factor expression in *Streptococcus pyogenes *[8]. Also, the *mcra *gene was up-regulated by the *mgrA *regulon in *Stapylococcus aureus*. This regulon has been shown to affect multiple *Staphylococcus aureus *genes involved in virulence and antibiotic resistance, but also genes involved in osmotic stress response (*opuD *(N315-SA1987, encoding the glycine betaine transporter), *proP *(N315-SA0531, encoding a proline/betaine transport homolog) and *gbsA *(SA2406, encoding glycine betaine aldehyde dehydrogenase)) [9].

The MCRA protein shows over 50% homology with linoleate isomerases from *Lactobacillus acidophilus *(Genbank: ABB43 157.1) and *Lb. reuteri *PYR8 linoleate isomerase [10], a protein that catalyses the formation of (9Z, 11E)-CLA from linoleic acid. CLA refers to several positional (9,11; 10,12; 11,13; etc.) and geometric (*cis *or *trans*) isomers of linoleic acid C18:2 *cis*-9 (*c*9), *cis*-12 (*c*12) octadecadienoic acid] with conjugated double bonds, and the (9Z, 11E) and (10E, 12Z) isomers in particular, have been shown to exert beneficial effects on human metabolism, including anti- carcinogenic, anti-diabetogenic and anti-atherogenic activities and body composition alteration effects. CLA is formed via biotransformation of linoleic acid, as a direct result of the action of the ruminal microbiota, of which *Butyrivibrio fibrisolvens *is the foremost [11-13]. The mechanism of microbial production of CLA was characterised using washed cells of the strain *Lb. acidophilus *AKU 1137 [14] and involves the production of hydroxy fatty acids as precursor to formation of CLA. When isolated and introduced to the washed cells, these hydroxy fatty acids were rapidly transformed to their respective CLA isomers. Thus, CLA formation by *Lb. acidophilus *was found to consist of two distinct steps, *step one: *the hydration of linoleic acid to 10-hydroxy-18:1 and *step two: *the subsequent dehydration and isomerisation of these hydroxy fatty acids to the *c*9,*t*11 CLA and *t*9,*t*11 CLA isomers.

We have previously reported the ability of a range of *Bifidobacterium *spp. to produce the *c*9,*t*11 CLA isomer from free linoleic acid [15-17] and have shown that administration of CLA producing *B. breve *NCIMB 702258 to mice and pigs in combination with linoleic acid alters host tissue fatty acid composition [18], including elevated liver *c*9,*t*11 CLA, coupled with reductions in the proinflammatory cytokines tumour necrosis factor-α (TNF-α) and interferon-γ (IFN-γ). The enzyme responsible for CLA production in *Bifidobacterium *has not been reported. The aims of this study were 1) to investigate if the MCRA protein from *B. breve *NCIMB 702258 was active as a fatty acid hydratase, by introducing the gene encoding MCRA into *Corynebacterium glutamicum *and *Lactococcus lactis *and analysis of the enzymatic activity of the expressed protein in the heterologous hosts and 2) to investigate whether this led to increased stress tolerance in the new hosts, and thus we report a role for MCRA in stress protection in *B. breve*.

## Methods

### Cultures and media

*B. breve *NCIMB 702258 was cultured in MRS media (pH 6.0, Oxoid Ltd, Hampshire, UK) supplemented with 0.05% (w/v) L-cysteine hydrochloride (Sigma Chemical, 98% pure) under anaerobic conditions using anaerobic jars with 'Anaerocult A' gas packs (Merck, Darmstadt, Germany) at 37°C. *Lactococcus lactis *NZ9800 (a *L. lactis *NZ9700 derivative which does not produce nisin because of a deletion in the *nis*A gene, and contains the *nis*RK signal transduction genes integrated on the chromosome) was cultured at 30°C in M17 (Difco laboratories, USA) broth and/or agar containing glucose (0.5% w/v). *L. lactis *carrying the plasmid pNZ8048 was routinely cultured in the presence of chloramphenicol (5 μg/ml) as a selective marker. *Corynebacterium glutamicum *ATCC 13032 was cultured aerobically in BHI media (Merck, Darmstadt, Germany) at 37°C. *C. glutamicum *ATCC 13032 carrying the shuttle vector pCLIK 5a MCS Pddh (BASF, Ludwigshafen, Germany) was routinely cultured in the presence of kanamycin (20 μg/ml) as a selective marker. *Escherichia coli *JM109(DE3) carrying the plasmid pOXO4 was routinely cultured aerobically in BHI at 37°C in the presence of chloramphenicol (25 μg/ml) as a selective marker.

### DNA sequence analysis and primer design

**Figure 1:**
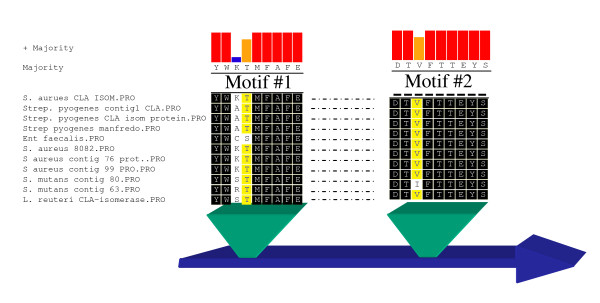
**Motifs used to design forward and reverse primers targeted to *B. breve *NCIMB 702258 *mcra *gene**.

The sequence encoding the 67 kDa MCRA (linoleate isomerase) protein from *Lb. reuteri *PYR8 [19] was compared to sequence databases (GenBank and unfinished genomes databases), using the BLAST suite of programs [20]. Proteins exhibiting significant similarity were aligned using DNAStar software (DNAStar Inc. Madison, WI, USA) and conserved motifs were identified. Degenerate oligonucleotide primers and primers specific for bifidobacteria (considering bifidobacteria codon usage) were designed to these motifs (YWXTMF AFE and DTVFTTEYS, Figure 1) and used in PCR reactions to amplify a ~1 kb internal region of the sequence as follows: Primer EV1a forward and EV2a reverse general, degenerate): 5'- TA(C/T),TGG,III,ACI,ATG,TT(C/T),GCI,TT(C/T),GA(A/G)-3' and 5'- GA,GA(T/A),(T/C)TC,IGT,IGT,(G/A)AA,IAC,IGT,(G/A)TC 3', EV1b forward and EV2b reverse (specific for bifidobacteria codon usage): 5'-TAC,TGG,III,ACC,ATG,TTC,GCI,TTC,GAA-3' and 5'- GA,GTA,TTC,(G/A)GT,(G/A)GT,GAA,GAC,(G/A)GT,(G/A)TC 3'. PCR reactions were performed in a Hybaid PCR Express Unit (Hybaid Ltd. Middlesex, UK) in 50 μl with an annealing temperature of 45°C. Each reaction contained 1 μl of each primer (50 pmol/μl), 2 μl of template in 5 μl MgCl2 (50 mM), 5 μl dNTP Master Mix (12.5 mM), 50 μl 10x NH4 Reaction Buffer and 0.5 μl Biotaq DNA Polymerase (5 U/μl) (BIOLINE, London, UK). The resulting ~1 kb fragment was cloned into pCR 2.1- TOPO and sequenced (MWG, Germany).

### Chromosome walking by inverse PCR

To obtain flanking chromosomal sequence, two primers were designed to terminal regions of the known chromosomal DNA sequence (Additional file 1). The genomic DNA from *B. breve *NCIMB 702258 was digested with a range of restriction enzymes followed by ligation with DNA ligase as described by the supplier (New England BioLabs Inc. Hertfordshire, UK). The circularized fragments were subsequently used as templates in the inverse PCR reactions with the terminal primers. The reactions were performed in the same way as the standard PCR reactions but with an annealing temperature of 50°C and the resulting fragments (analysed after separation by agarose gel electrophoresis) were cloned into the pCR2.1-TOPOvector and sequenced. The two terminal primers used were Primer A (upstream): 3'-CGTTCTCGACCTTGGTGTTGTATCGGAATT 5' and Primer B (downstream): 5' -GTACCGACCGACAAGATCGAGTCGCTTGCC-3'. From the approach described above, it was confirmed by sequencing that the 3' end of the gene was obtained from re-circularized *Xba*I digests; however the 5' end was not yet acquired. A second round of inverse PCR was performed with primers A (upstream) (3'- CGTTCTCGACCTTGGTGTTGTATCGGAATT-5') and pCS0 1 (downstream) (5' GCATCGATGTCAGCCAGG-3') targeted to re-circularized *Kpn*I digests of genomic DNA, which provided another 370 bp of the upstream region of the gene. The last round of inverse PCR to obtain the missing 170 bp from the 5' end of the gene was carried out with primers ER001 (5'-TCGATGGAGGGAATCGAA-3') and ER002 (5'-TATTGGCTCAACAAGGAAGAT-3') targeted to circularized *Hae*II digests.

### DNA manipulations and plasmid construction

Genomic DNA was isolated from *B. breve *NCIMB 702258 by a modified method described by Hoffmann *et al. *(1987) [21]. The 1878 *mcra *gene from *B. breve *NCIMB 702258 (Genbank: HQ593838) was amplified from genomic DNA with primers EMY01 and EMY02 (Table 1). PCR was performed with High Fidelity Expand Polymerase as described by the supplier (Roche Diagnostics, England) using 200 ng genomic DNA as a template. PCR conditions were as follows: 10 cycles of 2 min, 15 s denaturation (94°C), 30 s annealing (55°C), 2 min elongation (72°C) followed by 20 cycles of 15 s (94°C), 30 s (55°C), 2 min + 5 s/cycle (72°C) and finally, one 7 min cycle at 72°C.

**Table 1:** Primers used in this work. Restriction sites are underlined

Primer	Restriction site	Resulting construct	Sequence
EMY01	PstI	pEMYL1	5 '- AACTGCAGTGGATTGTCAATTCATCCCC- 3 '
			
EMY02	HindIII		5' - CCCCAAGCTTGCCCGATTATGCGAACGGCT- 3'
			
			

EMY03	NdeI	pEMYC2	5 '- AACATATGTGGATTGTCAATTCATCCCC- 3 '
			
EMY04	AvrII		5' - CCCCCCTAGGGCCCGATTATGCGAACGGCT- 3'
			
			

BBI_fw2	NdeI	pBBI2	5 ' - AACATATGTACTACAGCAGCGGCAACTATGAG- 3 '
			
BBI_rv2	XhoI		5 ' - TCTCGAGTTAGATCACATGGTATTCGCGTAGCAGGGTAGC- 3 '
			
			

The *mcra *gene was amplified with primers EMY03 and EMY04 (Table 1) for cloning in pCLIK, resulting in the construct pEMYC2 which was transformed into *C. glutamicum*. Recombinant plasmids were double digested with the same enzymes to verify the correct clone and electroporated into *L. lactis *NZ9 800 and *C. glutamicum *ATCC 13032. Electrocompetent *L. lactis *were prepared and transformed according to the method described by de Ruyter *et al. *(1996) [22], while electrocompetent *C. glutamicum *cells were prepared as described by Luchansky *et al. *(1988) [23]. For enzymatic assays, the recombinant *B. breve *MCRA protein was produced in *E. coli *as N-terminal 6x His-tag fusion. The complete *mcra *gene was amplified using primers BBI_fw2 and BBI_rv2 (Table 1). Ligation with pET28 vector (Novagen) resulted in a construct pBBI2 containing N-terminal fusion of 6x His tag and *B. breve *MCRA protein. The authenticity of the clones was verified by sequencing (MWG-BIOTECH, Germany).

### Investigation of the stress tolerance of MCRA protein-overproducing *L. lactis *and *C. glutamicum*

The MCRA protein was expressed in lactococci using the NICE system [22], which allows maximal protein expression at sub-lethal concentrations of nisin, and is tightly controlled, such that there is negligible expression in the absence of nisin [24]. The MCRA protein was also expressed in *C. glutamicum *using the shuttle vector pCLIK 5a MCS Pddh. This vector replicates in both *E. coli *and *C. glutamicum *and contains a *C. glutamicum *promoter (ddh promoter) and a kanamycin resistance gene which function in both *E. coli *and *C. glutamicum*. *L. lactis *pNZ8048 and pEMYL1 cells were induced with nisin (10 ng/ml) at OD600 nm ~ 0.5 and cultures were left to grow for an additional 1.5 h prior to heat treatment. *C. glutamicum *pCLIK and pEMYC2 were grown to OD600 nm ~ 0.4 - 0.5, prior to stress. Thermotolerance of *L. lactis *and *C. glutamicum *was monitored at 51°C in broth over 30-40 min. Aliquots were removed at 0, 10, 20, 30 and 40 min, plated on agar and incubated at 30°C (*L. lactis*) and 37°C (*C. glutamicum*) for 72 h. Salt tolerance was determined following resuspension in broth containing 3 M NaCl (*L. lactis*) and 4.8 M NaCl (*C. glutamicum*) and culture viability was monitored over 60-90 min with aliquots taken at 15 min intervals. Solvent tolerance was determined on addition of butanol 1-5% (v/v), and survival was monitored over 90 min with aliquots taken at 15 min intervals.

### Heterologous expression, protein extraction and SDS-PAGE

The putative linoleic acid isomerase was expressed in pOXO4 in *E. coli *using the T7 system (pOXO4 is a T7 RNA polymerase dependent expression plasmid). *E. coli *pOXO4 (control) and *E. coli *pEMYE3 cultures were aerated vigorously until the OD600_nm _reached approximately 0.6, at which point isopropyl-β-D-thiogalactopyranoside (IPTG) was added to final concentration of 0.5 mM to induce expression of the T7 RNA polymerase. Aliquots were taken at intervals after IPTG induction at 1, 3, 6 and 12 h for whole cell protein extraction and sodium dodecyl sulfate-polyacrylamide gel electrophoresis (SDS-PAGE). Similarly, *L. lactis *pEMYL1 and pNZ8048 (vector control) were cultured to OD600_nm _~0.5 and induced with nisin (10 ng/ml) and allowed to grow for another 12 h prior to protein extraction. *C. glutamicum *pEMYC2 and pCLIK (vector control) were cultured as described previously for 24 h prior to protein extraction. To extract proteins, cells (9 ml) were harvested and washed in 20 mM Tris HCl, pH 7.5 and resuspended in 0.5 ml extraction solution (20 mM Tris-HCl, pH 7.5, 8 M Urea and Chaps (0.5% w/v)) and bead-beaten for 2 × 30 sec with 1 min rest on ice. The cell lysate was then centrifuged to remove beads and cell debris and the protein extracts analysed by SDS-PAGE using a Mini Protean II cell unit (Bio-Rad) by the method of Laemmli [25] with a 10% acrylamide resolving gel and 4% stacking gel. A low molecular weight standard ranging from 14,400 to 97,400 Da (BioRad) was used as a molecular weight marker.

### Production and purification of recombinant *B. breve *MCRA protein from *E. coli*

For protein production, *E. coli *BL21 Star strain (Invitrogen, Darmstadt Germany) harboring pBBI2 plasmid was used. Bacteria were cultivated in 2xYT medium (tryptone (30 g/L), yeast extract (15 g/L), NaCl (5 g/L)) supplied with kanamycin (25 μg/ml) at 37°C until OD600 reached 0.6. At that point, IPTG was added to final concentration of 0.1 mM, the cells were changed to 16°C and harvested by centrifugation (10 min at 9100 g) after 18 h induction time. Cells were resuspended in buffer A (0.1 M Tris/HCl, pH 8.0, 0.15 M NaCl) in 1:3 v/v ratio, frozen in liquid nitrogen and stored at -20°C.

For protein purification, cells were lysed by adding lysozyme (0.1 mg/ml) and DNaseI (1 μg/ml) in buffer A and 10 mM MgCl2 and centrifuged for 20 min at 70,000 g. The supernatant was loaded on HisTrap column (GE Healthcare, Sweden), the column was washed with 10 column volumes of buffer A, and the protein was eluted with 3 column volumes of buffer A and 0.5 M imidazol. After elution, the buffer was exchanged to buffer B (0.1 M Tris/HCl, pH 7.5, 150 mM NaCl) by ultrafiltration and protein concentration was estimated spectrophotometrically using calculated absorption coefficient ε280 = 116990.

### Immunoblotting

10 μg of protein were subjected to SDS PAGE followed by protein transfer to nitrocellulose membrane at 60 V, 4 mA/cm^2 ^gel surface for 1 h. The membrane was washed with buffer B and blocked for one hour in buffer B containing 5% (w/v) milk powder. His-tagged proteins were detected using anti-His tag antibody (Sigma) in combination with a secondary antibody coupled with alkaline phosphatase and nitrotetrazolium blue/5-bromo-4-chloro-3-indolylphosphate staining.

### Spectroscopic assays

Solution of purified *B. breve *MCRA protein had yellow colour, therefore for initial characterization UV-Vis spectrum of purified protein in the range 300-600 nm was recorded with a single beam absorption spectrometer (Ultrospec 2100 *pro*, GE Healthcare). For the reference, spectra of FAD and FMN in Buffer B were recorded in the same way. For these measurements, *B. breve *MCRA protein, FAD and FMN were brought within the concentration range 10-40 pM. The absence of covalent bond between cofactor and *B. breve *MCRA protein was confirmed in the following way; 200 pl of the protein (10 mg/ml in 50 mM Tris/HCl, pH 7.5) was boiled for 10 min at 100°C and centrifuged at 12,000 g for 10 min. The spectrum of the supernatant was then recorded as described above.

### Activity assay, lipid extraction and derivatization

To demonstrate the enzymatic activity of *B. breve *MCRA protein, a panel of free fatty acids was tested in the reactions with the purified protein. Typically, 10-50 pg of substrate was mixed with 10 pg of purified protein in 1 mL of buffer B and incubated for 1 h at 25°C. Fatty acids were extracted from the reaction mixture with 1 ml chloroform:methanol (1:1) according to Bligh and Dyer [26]. Free fatty acids were dissolved in methanol and converted to corresponding methyl esters with (trimethylsilyl)-diazomethane. For GC- MS analysis, fatty acid methyl esters were dissolved in acetonitrile and hydroxy groups were modified by N,O-bis-(trimethylsilyl)-trifluoroacetamide (BSTFA), resulting in fatty acid trimethylsilylethers. GC-MS analysis was performed as previously described [27].

### Phylogenetic analysis

Homologs to the previously identified linoleate isomerase from *Lb. acidophilus *(Genbank: ABB43 157.1) and oleate hydratase, *Elizabethkingia meningoseptica *(Genbank: GQ144652) were selected from BLAST analysis. These homologs, along with MCRA from *B. breve *NCIMB 702258 discussed in this manuscript were aligned using T-Coffee [28] and protein trees were built using the PHYLIP package [29]. The cladogram was then visualised using DENDROSCOPE [30].

## Results

Previous studies by Rosson *et al. *[10] reported on the isolation and purification of a putative linoleic acid isomerase from *Lb. reuteri*. In these studies, protein sequencing had identified a 67 kDa protein with homology to MCRA. Based on this finding, we designed primers to conserved regions shared by a range of MCRA proteins in the database (Figure 1) and found that we could PCR the gene from a range of different *Bifidobacterium *strains and *Propionibacterium freudenreichii shermanii *(Additional file 2). Given that *B. breve *NCIMB 702258 is known to be capable of producing *c*9,*t*11 CLA from linoleic acid [16], we decided to study this strain in terms of CLA production. Using primers EV1a forward and EV2a reverse, we amplified a 1 kb internal fragment of the putative isomerase gene from *B. breve*. Sequencing of this revealed up to 70% homology to MCRA proteins from various genera. Following this, we primer walked in both 5'and 3' directions, which resulted in the full sequence of *B. breve *putative isomerase (Additional file 1).

### Sequence analysis

The complete 1878 bp nucleotide sequence from *B. breve *NCIMB 702258 encodes a protein of 626 amino acids with homology to other "67 kilodalton MCRA" proteins. The calculated molecular weight of the protein is 70535.38 Da. Comparison with sequences in the database revealed that the cloned MCRA protein showed 71% homology with putative 67 kDa MCRA from *Streptococcus mutans *UA159 (Genbank: NP_720953) and 51% homology with *Lb. acidophilus *linoleate isomerase (Genbank: ABB43 157.1). A non-canonical flavin binding motif (GxGxxA(x)21E) is present at the N-terminus of *B. breve *MCRA protein, similar to *Streptococcus *and *Lactobacillus *MCRA proteins [31]. The protein is cytoplasmic, but may have one trans-membrane helix as predicted by PSORTb (http://www.psort.org/). Upstream of the ORF is a putative dehydrogenase or reductase protein with ~50% homology with *B. longum *NCC2705 2,5-diketo-D-gluconic acid reductase (Genbank: AAN24097). Downstream of the TGA stop codon is a region of dyad symmetry, capable of forming a stable stem-and-loop structure. The potential hairpin consists of 13-bp stem separated by a 3-bp mis-priming and a 5- bp loop (ΔG, -15.01 kcal).

### Over-expression of *B. breve *MCRA protein in *E. coli*

Since no over-expression of the putative linoleic acid isomerase could be confirmed following SDS-PAGE of the recombinant lactococci and corynebacteria, possibly due to masking of the protein by other proteins of similar molecular weight (Additional file 3), the putative *lai *(linoleic acid isomerase) gene was expressed in pOXO4 in *E. coli *using the T7 system (pOXO4 is a T7 RNA polymerase dependent expression plasmid). Following growth and induction at OD600_nm _0.6 with 0.5 mM IPTG, aliquots of *E. coli *pOXO4 (control) and *E. coli *pEMYE3 were taken at 1, 3, 6 and 12 h after induction for whole cell protein extraction and SDS-PAGE. A protein band of approximately 66 kDa was visualized, corresponding to the expected molecular weight of the putative isomerase protein, which was absent in the IPTG induced control culture *E. coli *pOXO4 (Additional file 3). Following incubation for 48-72 h with free linoleic acid (0.5 mg/ml), the substrate for bacterial conversion to *c*9,*t*11 CLA, no isomerase activity could be confirmed among the heterologous clones carrying the *lai *gene in the bacterial supernatant by GC (Additional file 4), or by measuring OD234 _nm _[17].

### Production of *B. breve *MCRA in *E. coli. *and enzymatic assays

**Figure 2:**
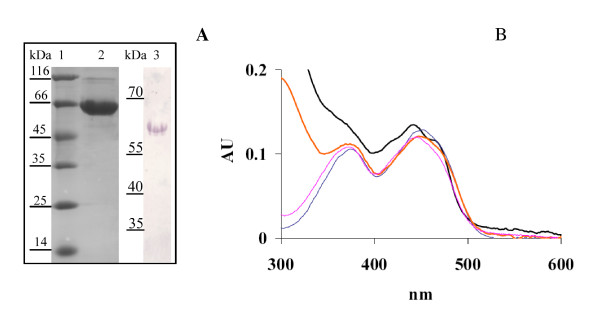
**Spectral analysis of purified *B. breve *MCRA protein**. SDS PAGE analysis of *B. breve *MCRA protein purified on Ni-NTA column. Lane 1, molecular weight marker, lane 2, *B. breve *MCRA protein of apparent MW of 66 kDa, lane 3, immunoblot with anti-His tag antibody shows the absence of significant proteolytic degradation in purified *B. breve *MCRA. B UV-Vis spectrum of *B. breve *MCRA protein (black) and the supernatant after heat precipitation (red), showing flavin-like absorbance. For the reference spectra of 10 μM FAD (blue) and FMN (magenta) are shown.

**Figure 3:**
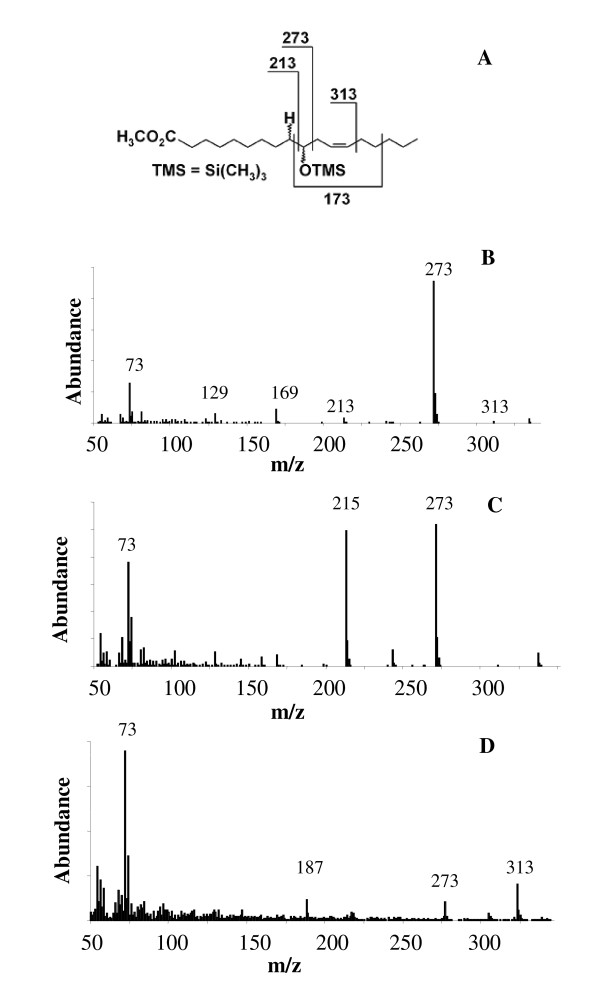
**Mass spectra of reaction products from the incubations of *B. breve *MCRA protein with free fatty acids**. A) Fragmentation pattern of 10-HOE. B) Mass spectrum of 10-HOE obtained after the incubation with linoleic acid. C) Mass spectrum of 10-HO obtained after the incubation with oleic acid. D) Mass spectrum of 10-HH obtained after the incubation with palmitoleic acid.

*B. breve *MCRA protein was overproduced in *E. coli *and purified on Ni^2+^-NTA column (Figure 2A, lane 2). The major band with apparent molecular weight of 66 kDa was visualized on SDS PAGE after the elution. Immunoblotting with anti-His tag antibodies confirmed that this band contained 6x His tag (Figure 2A, lane 3). Purified *B. breve *MCRA protein has flavin-like UV-Vis absorption spectrum with maxima around 450 and 370 nm (Figure 2B) and the flavin cofactor is released in the solution upon protein denaturation by heat. The spectral properties of *B. breve *MCRA are consistent with a recombinant MCRA protein from *S. pyogenes*, which was found to contain a non-covalently bound FAD and exhibited double bond hydratase activity producing 10- and 13-hydroxy fatty acids [1]. These data prompted us to examine the enzymatic activity of *B. breve *MCRA using common C16 and C18 fatty acids as substrates. To this end, purified protein was incubated with free fatty acids and the reaction products were analyzed by GC-MS. Indeed, we observed conversion of palmitoleic, oleic and linoleic acids to the corresponding 10-hydroxy fatty acids (Figure 3 and Table 2), but myristoleic acid or shorter monounsaturated fatty acids were not substrates. The presence of trans-double bonds as well as the double bonds beyond the position C12 abolished hydratase activity. No conversion of esterified fatty acids such as methyl linoleate or CLA isomers was observed.

**Table 2:** Reaction products of recombinant purified *B. breve *MCRA protein

Substrate	Product	RT, min	MS fragments, m/z
14: 1^A9^^*Z*^	No products	-	-

16:1^A9^^*Z *^(PA)	10-HH	8.13	357(M-1)^+^, 273, 187, 169

18:1^A9^^*Z *^(OA)	10-HO	9.56	385(M-1)^+^, 273, 215, 169

18:1^A11^^*Z*^	No products	-	-

18: 1^A9^^*E*^	No products	-	-

18:2^A9^^*Z*^^,12^^*Z *^(LA)	10-HOE	9.84	383(M-1)^+^, 273, 213, 173, 169

18:2^A9^^*Z*^^,A12^^*Z*^			

No products		-	-

methyl ester			

CLA mixture	No products	-	-

18:2^A9^^*E*^^,12^^*E*^	No products	-	-

18: ^3A9^^*Z*^^,12^^*Z*^^,15^^*Z *^(LeA)	No products	-	-

20:4A5*Z*,8*Z*,11*Z*,14*Z *(AA)	No products	-	-

### Phylogenetic analysis

**Figure 4:**
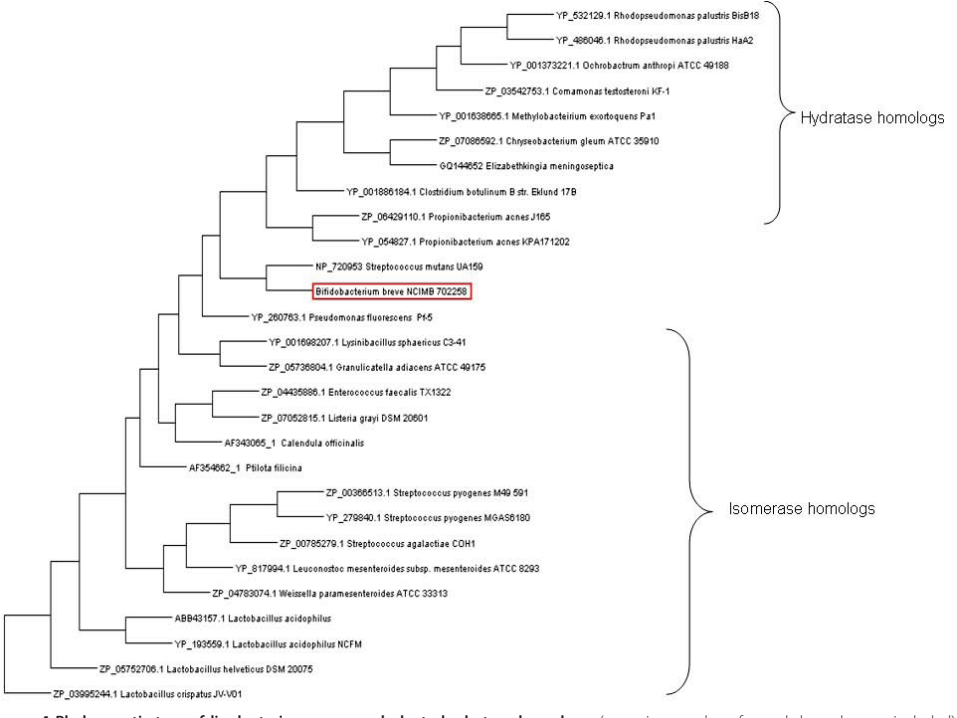
**Phylogenetic tree of linoleate isomerase and oleate hydratase homologs**. (accession numbers for each homolog are included). The placement of MCRA protein from *B. breve *NCIMB 702258 is highlighted in red. All branches are supported at >75% bootstrap values.

Figure 4 displays a phylogenetic analysis of MCRA from *B. breve *NCIMB 702258 along with both linoleate isomerase and oleate hydratase homologs. The tree clearly illustrates distinct groupings of the two sets of homologs. Interestingly, our MCRA protein branches in between both groupings but appears to be evolutionary closer to the hydratase group. This result further supports our finding of the MCRA as fatty acid hydratase.

### Stress tolerance of MCRA-overproducing *Lactococcus *and *Corynebacterium*

**Figure 5:**
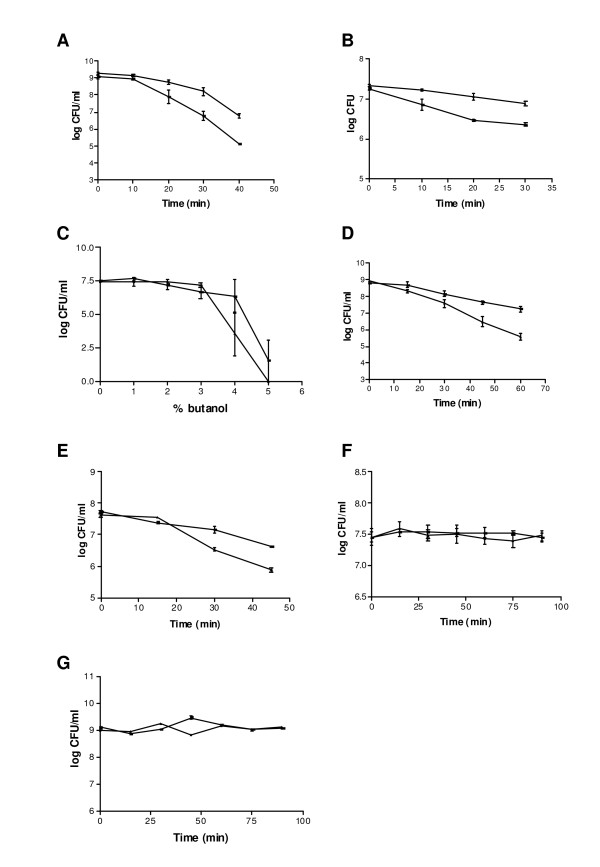
**Effect of *B. breve *MCRA expression on survivability of *L. lactis *and *C. glutamicum *strains under stress conditions**. A) Survivability of *L. lactis *pNZ8048 ('black square') versus pEMYL1 ('upside down black triangle') following heat treatment at 51°C for 40 min. ** Denotes value significantly different (*p = *0.01) than control (pNZ8048). B) Survivability of *C. glutamicum *pCLIK ('black square') versus pEMYC2 ('black triangle') following heat treatment at 51°C for 30 min * Denotes value significantly different (*p = *0.05) than control (*C. glutamicum *pCLIK). C) Survivability of *C. glutamicum *pCLIK ('upside down black triangle') versus pEMYC2 ('black square') following growth in 1-5% (v/v) butanol for 1 h. D) Survivability of *L. lactis *pNZ8048 ('black triangle') versus pEMYL1 ('black square') following nisin induction for 1.5 h prior to growth in 3% (v/v) butanol for 1 h. The experiments were done at least in triplicate. * Denotes value significantly different (*p = *0.05) than control (pNZ8048). E) Survivability of *C. glutamicum *pCLIK ('upside down black triangle')versus pEMYC2 ('black square') following growth in 3% (v/v) butanol for 45 min. F) Survivability of *C. glutamicum *pCLIK ('black triangle')versus pEMYC2 ('black square') following growth in 4.8 M NaCl for 90 min. G) Survivability of *L. lactis *pNZ8048 ('black triangle') versus pEMYL1 ('black square') following nisin induction for 1.5 h prior to growth in 3 M NaCl for 90 min. The experiments were done in triplicate.

To determine if heterologous *Lactococcus *and *Corynebacterium *containing the *mcra *gene exhibited improved stress tolerance, the heterologous cultures along with vector controls of *Lactococcus *(pNZ8048) and *Corynebacterium *(pCLIK) were assayed for hydratase activity by measuring 9-hydroxy fatty acids without and with linoleic acid supplementation of the medium. Indeed, those cultures that expressed MCRA/hydratase accumulated increasing amounts of 10-HOA and 10-HOE in the culture medium (Additional file 5). Next, the heterologous cultures were exposed to heat (51°C × 30-40 min), salt (3 M NaCl × 90 min - *Lactococcus *and 4.8 M NaCl × 90 min - *Corynebacterium*) and solvent (3% (v/v) butanol × 45-60 min) stresses. *L. lactis *pNZ8048 reduced in viability by 4 Log CFU/ml following heat stress at 51°C for 40 min, whereas *L. lactis *pEMYL1 only reduced in viability by 2 Log CFU/ml (Figure 5A, B). The difference in survivability between *C. glutamicum *pCLIK and pEMYC2 was ~0.5 Log CFU/ml when heat stressed at 51°C for 30 min, a decline in survivability by <0.5 Log CFU/ml for *L. lactis *pEMYL1 and <1 Log CFU/ml for *C. glutamicum *pCLIK (vector control).

Similarly, when induced *L. lactis *pNZ8048 was exposed to solvent stress (3% (v/v) butanol), a decrease in viability of >3.3 Log CFU/ml was observed, whereas induced *L. lactis *pEMYL1 only decreased ~1.5 Log CFU/ml following 60 min in 3% (v/v) butanol, i.e. a difference of nearly 2 Log CFU/ml between the cultures under identical conditions. *C. glutamicum *pEMYC2 exposed to 3% (v/v) butanol stress for 45 min survived ~ 0.75 Log CFU/ml better than the vector control (pCLIK) (Figure 5C-E).

Upon confirming that the clones containing the MCRA protein were less sensitive to heat and solvent stresses, we examined the hypothesis that the protein also protected the cultures from salt stress. Following growth and induction by nisin (10 ng/ml at OD600 nm ~0.5) and additional growth for 1.5 h, *L. lactis *pNZ8048 (control) and pEMYL1 cultures were exposed to 3 M NaCl for 90 min. However, no difference in salt tolerance was observed at concentrations of 3 M NaCl. A similar observation was made for *C. glutamicum *pCLIK and pEMYC2 which were grown in 4.8 M NaCl for 90 min. Both *C. glutamicum *control (pCLIK) and pEMYC2 cultures survived in 4.8 M NaCl for 90 min with no apparent decline in viable numbers (Figure 5F, G).

## Discussion

Several recent reports have linked MCRA and oleic acid hydratase activity [1-3], which was first observed more than forty years ago in *Pseudomonas *sp. [32]. However, the significance of this hydratase activity for the bacterial host is not yet clearly established. Our study identified MCRA from *B. breve *as a FAD-containing hydratase with an additional role in stress protection (Figures 2 and 3, Table 2). Interestingly, a recent study by O'Flaherty and Klaenhammer (2010) indicated a role for MCRA in stress tolerance, cell wall division and adherence to Caco-2 cells [33]. Furthermore, all three characterized enzymes from this family are highly specific for free fatty acids as the previously characterized linoleic acid isomerase from *P. acnes *[1,2,34]. Thus, both enzyme activities seem to form a new class of FAD-dependent fatty acid modifying enzymes. This finding may be even further supported by phylogenetic groupings (Figure 4). Given the wide distribution of the *mcra *gene between human pathogenic and commensal bacteria, it could be speculated that it is not directly linked to virulence but to a general stress protection, which could also be useful for a pathogen i.e. increased heat at the site of infection. Our study confirmed the increased viability of heterologous *Lactococcus *and *Corynebacterium *transformed with the *mcra *gene during heat and solvent stresses as a difference in viability of 2 Log CFU/ml following heat stress at 51°C for 40 min and 3% (v/v) butanol stress for 60 min was observed between *L. lactis *pEMYL1 and pNZ8048 (Figure 4A-E). Likewise, the introduced MCRA improved the survivability of *C. glutamicum *by ~0.5 Log CFU/ml difference between *C. glutamicum *pCLIK and pEMYL1 following heat stress at 51°C for 30 min, and ~0.75 Log CFU/ml following 3% (v/v) butanol stress for 45 min (Figure 5).

The same host, *L. lactis *NZ9800 has previously been used for comparative studies of stress performance following transformation with the well known heat shock protein GroESL in plasmid pNZ8048. Desmond *et al. *(2004) [35] compared the viability of induced (nisin 10 ng/ml) and heat treated cells at 54°C for 30 min of the same host *L. lactis *NZ9800 transformed with either pNZ8048 or pNZ8048-GroESL. At that temperature, both *L. lactis *pNZ8048 and un-induced *L. lactis *pNZ8048-GroESL (pGRO1) reduced in viability by 4.0 log CFU/ml, whereas the induced *L. lactis *(pGRO1) declined by 3.3 Log CFU/ml during the heat stress. Furthermore, induced GroESL-overproducing *L. lactis *(pGRO1) was able to grow and increase by 0.5 Log CFU/ml during 5 h exposure to 0.5% (v/v) butanol, compared with *L. lactis *(pNZ8048) and un-induced *L. lactis *(pGRO1) which declined in viability after 1 h ( 0.2 Log CFU/ml), a trend that continued over the 6 h period of solvent challenge.

It has been reported that the response of cells to sublethal heat and ethanol exposure induce essentially identical stress responses because of the alteration in the membrane fluidity by these stresses [36]. Heat and ethanol stresses cause similar changes to plasma membrane protein composition, reducing the levels of plasma membrane H^+^- ATPase protein and inducing the plasma membrane-associated Hsp30. Both stresses also stimulate the activity of the fraction of H^+^-ATPase remaining in the plasma membrane. The resulting enhancement to catalysed proton efflux from the cell represents a considerable energy demand, yet may help to counteract the adverse effects for homeostasis of the increased membrane permeability that results from stress.

## Conclusions

This study has shown that the MCRA protein in bifidobacteria is a FAD containing hydratase enzyme, which may be catalysing the first step in CLA production in this species, and has an additional function in bacterial stress protection.

## Abbreviations

10,13-DiHOA: 10,13-dihydroxy octadecanoic acid; FAD: flavin adenine dinucleotide; FMN: flavin adenine mononucleotide; HEPES: 4-(2-hydroxyethyl)- 1 -piperazine ethane-sulfonic acid; 10-HOA: 10-hydroxy octadecanoic acid; 10-HOE: (12Z)-10- hydroxy-12-octadecenoic acid; HPLC: high pressure liquid chromatography; MCRA: myosin cross-reactive antigen; MS: mass-spectroscopy; PAI: *Propionibacterium acnes *isomerase; Tris: 2-Amino-2-(hydroxymethyl)propane- 1, 3-diol,

## Authors' contributions

ER-C: collection and analysis of data, writing of manuscript, AL: collection and analysis of data, CG: collection and analysis of data RPR: design of experiments, editing of manuscript, OOS: collection and analysis of data, SFFF: design of experiments, GF: design of experiments IF: design of experiments, analysis of data and writing of manuscript, and CS: design of experiments, data analysis and editing of manuscript. All authors have read and approved the final manuscript.

## Supplementary Material

Additional file 1 **Map of putative isomerase gene**. Map of putative isomerase gene and chromosome walking strategy (inverse PCR) used to obtain the full sequence. Click here for file

Additional file 2 ***Mcra *gene sequence data from *Bifidobacterium *strains and *Propionibacterium freudenreichii shermanii***. 1 kb PCR fragments following PCR of genomic DNA from a range of bifidobacterial strains and *Propionibacterium freudenreichii shermanii *9093 using oligonucleotide primers EV1a forward and EV2a reverse. Lanes 1-12 in the following order; *B. adolescentis*; *B. breve *2257; *B. breve *702258; *B. breve *8815; *B. dentium *2243; *B. infantis *2205; *B. lactis *Bb 12; *B. longum *2259; *B. longum *BB 536; *Bifidobacterium *sp. 35612; *Bifidobacterium *sp. 35687; *Propionibacterium freudenreichii shermanii *9093. Click here for file

Additional file 3 **Putative linoleic acid isomerase protein**. Panel A. *E. coli *JM109(DE3) pOXO4 (lanes 1-4) and pEMYE3 (lanes 5- 8) induced with 0.5 mM IPTG. Samples taken after 1, 3, 6 and 12 h growth for protein extraction and SDS-PAGE. Panel B. *C. glutamicum *pCLIK (lane 1) and pMYC2 (lane 2) following 24 h growth prior to protein extraction. Panel C. *L. lactis *pNZ4848 (lanes 1-2) and pEMYL1 (lanes 3-4) following induction with nisin 0 and 10 ng/ml, respectively, and further growth for 12 h prior to protein extraction. Click here for file

Additional file 4 **CLA forming activity in bacterial strains expressing MCRA from *B. breve***. GC chromatogram of supernatant from *B. breve *NCIMB 702258 (positive control), *L. lactis *pEMYL1 (carrying *laiA *gene), and *L. lactis *pNZ8048 (vector control) following growth in linoleic acid (0.1 mg/ml) (*L. lactis*) and (0.5 mg/ml (*B. breve*) for 48-72 h. Similar results were obtained for recombinant *C. glutamicum *and *E. coli*. Click here for file

Additional file 5 **Hydratase activity in bacterial strains expressing MCRA from *B. breve***. Amounts of 10-HOA (black bars) and 10-HOE (gray bars) in supernatant from *L. lactis *pEMYL1 (carrying *MCRA *gene), and *L. lactis *pNZ8048 (VC, vector control) following growth in linoleic acid (0.1 mg/ml) (*L. lactis*) for 2-30 h. Data represent the mean values of 3 measurements. Similar results were obtained for recombinant *C. glutamicum*. Click here for file
