# Supplementary figures and images for: Myosin-cross-reactive antigen (MCRA) protein from Bifidobacterium breve is a FAD-dependent fatty acid hydratase which has a function in stress protection
Source: BMC Biochem. 2011 Feb 17;12:9. doi: 10.1186/1471-2091-12-9 (PMC3063827; doi:10.1186/1471-2091-12-9)

Suppl. Fig. 1


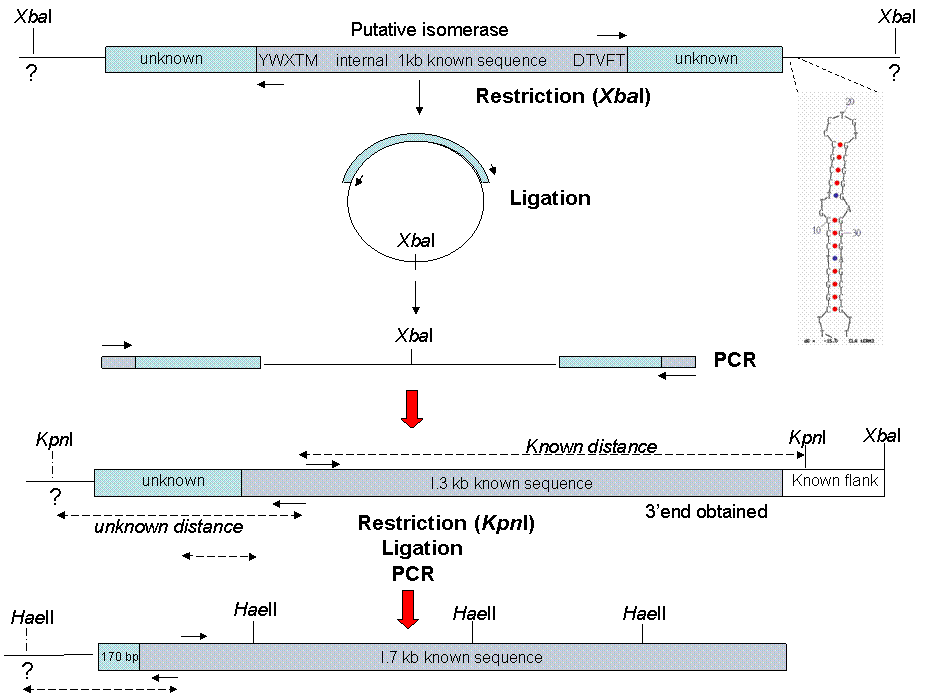

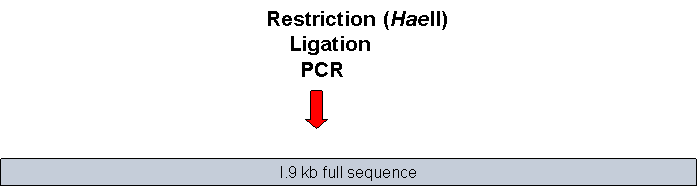

Supplement: Additional file 1 — Map of putative isomerase gene. Map of putative isomerase gene and chromosome walking strategy (inverse PCR) used to obtain the full sequence. [file 1471-2091-12-9-S1.doc]

Suppl. Fig. 2


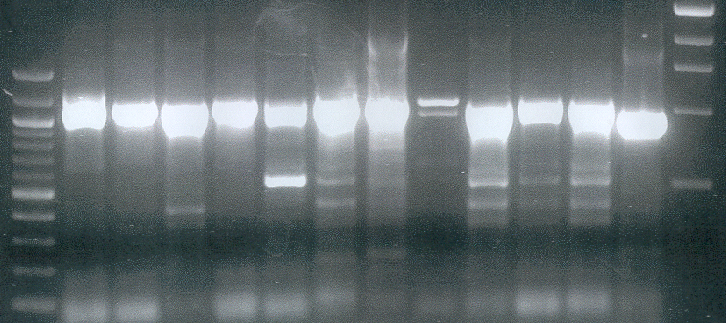


**1 kb**

Supplement: Additional file 2 — Mcra gene sequence data from Bifidobacterium strains and Propionibacterium freudenreichii shermanii. 1 kb PCR fragments following PCR of genomic DNA from a range of bifidobacterial strains and Propionibacterium freudenreichii shermanii 9093 using oligonucleotide primers EV1a forward and EV2a reverse. Lanes 1-12 in the following order; B. adolescentis; B. breve 2257; B. breve 702258; B. breve 8815; B. dentium 2243; B. infantis 2205; B. lactis Bb 12; B. longum 2259; B. longum BB 536; Bifidobacterium sp. 35612; Bifidobacterium sp. 35687; Propionibacterium freudenreichii shermanii 9093. [file 1471-2091-12-9-S2.doc]

Suppl. Fig. 3


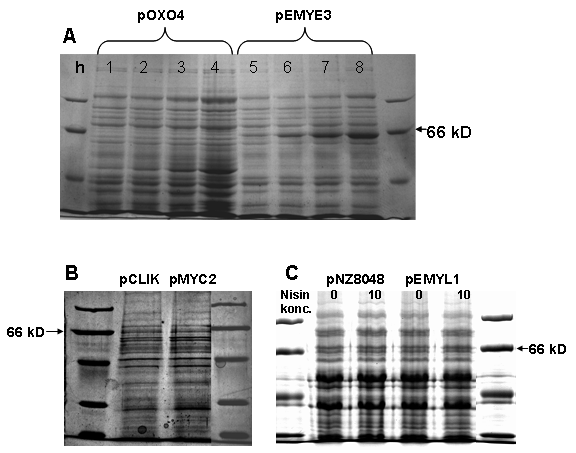

Supplement: Additional file 3 — Putative linoleic acid isomerase protein. Panel A. E. coli JM109(DE3) pOXO4 (lanes 1-4) and pEMYE3 (lanes 5- 8) induced with 0.5 mM IPTG. Samples taken after 1, 3, 6 and 12 h growth for protein extraction and SDS-PAGE. Panel B. C. glutamicum pCLIK (lane 1) and pMYC2 (lane 2) following 24 h growth prior to protein extraction. Panel C. L. lactis pNZ4848 (lanes 1-2) and pEMYL1 (lanes 3-4) following induction with nisin 0 and 10 ng/ml, respectively, and further growth for 12 h prior to protein extraction. [file 1471-2091-12-9-S3.doc]

Suppl. Fig. 5


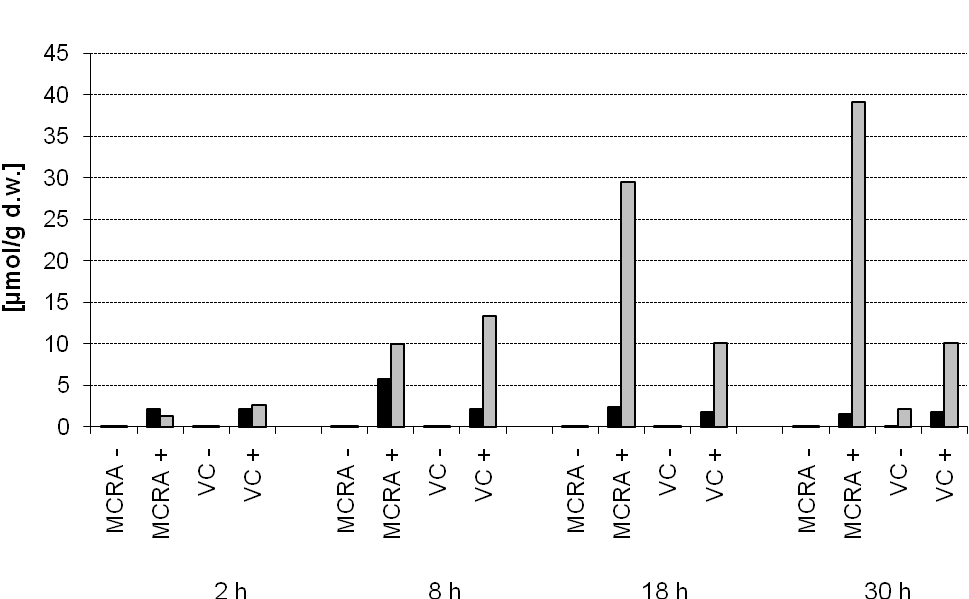

Supplement: Additional file 5 — Hydratase activity in bacterial strains expressing MCRA from B. breve. Amounts of 10-HOA (black bars) and 10-HOE (gray bars) in supernatant from L. lactis pEMYL1 (carrying MCRA gene), and L. lactis pNZ8048 (VC, vector control) following growth in linoleic acid (0.1 mg/ml) (L. lactis) for 2-30 h. Data represent the mean values of 3 measurements. Similar results were obtained for recombinant C. glutamicum. [file 1471-2091-12-9-S5.doc]
