# Supplementary material for: Myosin-cross-reactive antigen (MCRA) protein from Bifidobacterium breve is a FAD-dependent fatty acid hydratase which has a function in stress protection
Source: BMC Biochem. 2011 Feb 17;12:9. doi: 10.1186/1471-2091-12-9 (PMC3063827; doi:10.1186/1471-2091-12-9)
Supplement: Additional file 4 — CLA forming activity in bacterial strains expressing MCRA from B. breve. GC chromatogram of supernatant from B. breve NCIMB 702258 (positive control), L. lactis pEMYL1 (carrying laiA gene), and L. lactis pNZ8048 (vector control) following growth in linoleic acid (0.1 mg/ml) (L. lactis) and (0.5 mg/ml (B. breve) for 48-72 h. Similar results were obtained for recombinant C. glutamicum and E. coli. [file 1471-2091-12-9-S4.doc]

Suppl. Fig. 4
